# Predictors of blood ionized calcium concentration in sick adult cattle

**DOI:** 10.1111/jvim.16938

**Published:** 2023-12-01

**Authors:** Tolga Karapinar, Kenan Cagri Tumer, Peter D. Constable, Sébastien M. C. Buczinski

**Affiliations:** ^1^ Department of Internal Medicine, Faculty of Veterinary Medicine Firat University Elazig Turkey; ^2^ Department of Internal Medicine, Faculty of Veterinary Medicine Kastamonu University Kastamonu Turkey; ^3^ College of Veterinary Medicine University of Illinois Urbana‐Champaign Urbana Illinois USA; ^4^ Département des Sciences Cliniques Faculté de Médecine Vétérinaire, Université de Montréal Saint‐Hyacinthe Quebec Canada

**Keywords:** blood pH, hypochloremia, ionized calcium

## Abstract

**Background:**

Data on the factors affecting blood ionized calcium concentration (*c*iCa^2+^) and diagnostic performance of serum total calcium concentration (*c*tCa) measurements to detect abnormal blood iCa^2+^ status are lacking in sick adult cattle.

**Objective:**

Assess the association of *c*iCa^2+^ with venous blood pH, plasma concentrations of chloride (*c*Cl), sodium (*c*Na), and potassium (*c*K), and *c*tCa, and total protein, albumin, and globulin concentrations in sick adult cattle.

**Animals:**

Two‐hundred and sixty‐five adult cattle (≥1‐year‐old) with different diseases.

**Methods:**

Prospective study. Whole blood pH, *c*iCa^2+^, *c*Na, *c*K, and *c*Cl were measured using a blood gas and electrolyte analyzer, whereas *c*tCa, and total protein, and albumin concentrations were determined using an autoanalyzer. The relationship between *c*iCa^2+^ and venous blood pH, plasma *c*Cl, *c*Na, *c*K, and *c*tCa, and total protein, albumin, and globulin concentrations was investigated. Sensitivity and specificity were calculated for *c*tCa for diagnosis of abnormal *c*iCa^2+^.

**Results:**

Sensitivity of *c*tCa measurements to detect abnormal *c*iCa^2+^ was 66.0% whereas specificity of *c*tCa measurements was 72.3%. Serum total calcium concentration measurements accounted for 42% of adjusted blood ionized calcium (iCa^2+^_7.40_) concentration variance. Plasma *c*Cl, and *c*K had explanatory power of *c*iCa^2+^_7.40_, accounting for an additional 21% and 9% of the variance, respectively.

**Conclusions and Clinical Importance:**

Serum tCa measurements failed to accurately predict blood iCa^2+^ status in ill adult cattle. Serum tCa concentrations and plasma *c*Cl were the strongest predictors of *c*iCa^2+^ in sick adult cattle.

**Abbreviations**
Ca^2+^
: calcium
*c*Cl: chloride concentration
*c*iCa^2+^
: blood ionized calcium concentration
*c*iCa^2+^_7.40_
: blood iCa^2+^ concentration adjusted to pH = 7.40
*c*K: potassium concentration
*c*Na: sodium concentration
*c*tCa: serum total calcium concentration
CV: coefficient of variation
iCa^2+^
: ionized calcium
NLR: negative likelihood ratio
PLR: positive likelihood ratio
Se: sensitivity
SID: strong ion difference
Sp: specificity
tCa: total calcium
VIF: variation inflation factor

## 1 INTRODUCTION

Disorders of calcium (Ca^2+^), especially hypocalcemia, are important problems in bovine medicine.^1^, ^2^ Periparturient hypocalcemia may lead to various metabolic and infectious diseases in dairy cows, and hypocalcemia is a common clinicopathologic finding in cattle with systemic diseases or acute toxemic conditions.^1^, ^2^, ^3^ Calcium exists 3 different fractions in plasma or serum: protein‐bound calcium, ionized calcium (iCa^2+^), and complexed calcium.^1^, ^4^ Ionized Ca^2+^ is the free and biologically active form of Ca^2+^ in the blood. Although blood iCa^2+^ concentrations (*c*iCa^2+^) can be measured using conventional blood gas analyzers in veterinary hospitals, such measurements may be unavailable and technically challenging especially in farm conditions. Therefore, clinicians continue to rely on measurement of serum total calcium (tCa) concentration (*c*tCa) to assess calcium status in adult cattle.

Plasma iCa^2+^ is primarily dependent on *c*tCa, with *c*tCa explaining 64% to 86% of the variation in iCa^2+^ in plasma or serum from adult cattle.^5^, ^6^, ^7^ A recent study in critically ill calves indicated that plasma iCa^2+^ concentration was associated with plasma tCa, venous blood pH, plasma chloride (cCl), serum magnesium, and plasma L‐lactate (*R* ^2^ = 0.69) concentrations but not plasma albumin.^4^ Apart from tCa, pH has the next largest effect on the iCa^2+^ concentration of calf plasma^8^ and cow plasma,^9^ with pH‐corrective equations for plasma iCa^2+^ concentration being similar to those used for human plasma.^4^ Metabolic acidosis usually develops in ill neonatal calves, but hypochloremic metabolic alkalosis is a more common clinicopathologic finding in sick adult cattle.^3^ Different clinicopathologic findings between ill calves and sick adult cattle may affect blood iCa^2+^ concentration in a different manner. Therefore, the relative effect of blood pH and plasma *c*Cl and other electrolytes on blood iCa^2+^ needs to be accurately determined in sick adult cattle. In clinical settings, we commonly observed discrepancy between serum tCa and blood iCa^2+^ results. For this reason, we hypothesized that tCa concentration in sick adult cattle was not a good predictor of blood iCa^2+^ concentration. Our objectives were therefore to: (a) determine the relationship between serum tCa concentrations and *c*iCa^2+^ in adult cattle affected by various diseases, (b) investigate the association between venous blood pH, plasma *c*Cl, sodium (*c*Na), potassium (*c*K), and *c*tCa, and total protein, albumin, and globulin concentrations and *c*iCa^2+^ in sick adult cattle and (c) determine the percentage of blood iCa^2+^ fraction in serum tCa in adult cattle with different diseases.

## 2 MATERIALS AND METHODS

### 2.1 Animals

Two‐hundred and sixty‐five cattle (≥1‐year‐old) with different diseases referred to Firat University Teaching and Training Animal Hospital between January 2018 and April 2019 were recruited in this prospective study of a convenience sample. Sample size was calculated using R pwr package. Assuming a minimal coefficient of determination of the final multivariable model of 0.1 and a maximum of 10 different predictors, a minimal sample size of 193 samples was required (90% power, 5% type 1 error). Cattle with various systemic disorders were included in the study to obtain a wide range of venous blood pH and serum electrolyte, tCa, total protein, albumin, and globulin concentrations. Cattle that received calcium solutions within 48 hours before collecting blood samples were excluded from the study. The clinical diagnosis was obtained based on clinical diagnostic evaluation. When more than 1 disease was present, the main diagnosis was retained for that specific case for descriptive purposes. This study was approved by the Firat University Ethics Committee on Animal Experimentation (Permit number: 179).

### 2.2 Blood samples

Blood samples were anaerobically collected from the left or right jugular vein of cattle into blood gas syringes (Pico 50, Radiometer Medical ApS, Brønshøj, Denmark) containing 80 IU dry, electrolyte‐balanced heparin and plain tubes. The volume of the blood gas syringe was 0.5‐2 mL, and approximately 1.5 mL of a blood sample was aspirated into a syringe in all cases except for 2 cases in which 0.5 mL of blood sample was collected because of challenges in restraining the animal. Whole blood pH, *c*iCa^2+^, *c*Na, *c*K, and *c*Cl were measured within 10 minutes of blood collection using a Radiometer ABL80 analyzer. Venous blood pH was measured using a glass pH electrode, whereas *c*iCa^2+^, *c*Na, *c*K, and *c*Cl were measured using direct ion selective electrode technology. Values for venous blood pH corrected for rectal temperature were not used in this study. The strong ion difference (SID_3_) in mEq/L was calculated from the *c*Na, *c*K, and *c*Cl: SID_3_ = *c*Na + *c*K − *c*Cl.^10^ Hematocrit measurement of venous blood samples was performed once using capillary microhematocrit tubes after centrifugation for 5 minutes. Blood collected into plain tubes was allowed to clot and centrifuged at 1500×*g* for 15 minutes. Serum was harvested and stored −20°C until analyzed within 7 days of collection. Serum total protein (biuret), albumin (bromocresol green), and tCa (arsenazo dye binding) concentrations were measured using a traditional bench‐top autoanalyzer (SIEMENS Advia 1800, Siemens Healthcare GmbH, Erlangen, Germany). Serum globulin concentrations were calculated as the difference between serum total protein and albumin concentrations. The following equation was used to calculate the percentage of blood iCa^2+^ in tCa, iCa^2+^ percentage = [iCa^2+^ (mmol/L) × 100]/tCa (mmol/L), with mg/dL of serum tCa being multiplied by 0.2495 to convert into mmol/L. The intra‐assay coefficient of variation (CV) of the Radiometer ABL80 analyzer for venous blood pH and *c*iCa^2+^, *c*Na, *c*K, and *c*Cl measurements was determined from 8 measurements of the same sample during 1 day. The inter‐assay CV of the autoanalyzer for serum total protein, albumin, and tCa measurements was calculated from 10 stored sample aliquots from the same animal that were measured on 10 consecutive days.

### 2.3 Evaluation of serum total calcium concentration diagnostic performance

The diagnostic performance of *c*tCa was assessed for correctly predicting blood iCa^2+^ status. The reference interval for *c*iCa^2+^ was 1.06‐1.26 mmol/L.^2^, ^11^, ^12^ The reference range for *c*tCa concentration was 2.00‐2.60 mmol/L.^13^ Cattle were categorized as hypocalcemic, normocalcemic, or hypercalcemic on the basis of *c*iCa^2+^ and *c*tCa concentrations. Sensitivity (Se), specificity (Sp), negative likelihood ratio (NLR), and positive likelihood ratios (PLR) were calculated for *c*tCa for diagnosis of abnormal *c*iCa^2+^. Positive likelihood ratios >10 indicate that a positive test is good at diagnosis of abnormal *c*iCa^2+^, whereas NLR <0.1 indicates that a negative test is good at ruling out a diagnosis of abnormal *c*iCa^2+^.^14^, ^15^ Diagnostic discordance was determined by use of the following equation: diagnostic discordance = (number of samples with diagnostic disagreement between measured *c*iCa^2+^ and *c*tCa/total number of samples) x 100.^16^ Diagnostic performance of *c*tCa was calculated using MedCalc (software version 20.110, MedCalc, Ostend, Belgium).

### 2.4 Statistical analysis

Analyses were performed using the R open access statistical software (R Core Team [2020]. R: A language and environment for statistical computing. R Foundation for Statistical Computing, Vienna, Austria). Two different types of analyses were performed based on a previous study^4^ that proposed correcting ionized calcium to a pH of 7.40 using the equation:

$${\mathrm{iCa}}_{7.40}=\mathrm{iCa}\times 10^{\left(-0.23\times \left(7.4-{\mathrm{pH}}_{m}\right)\right)},$$

where pH_m_ is the venous blood pH of the cow. The same approach therefore was taken for determining the association between clinical variables and iCa^2+^ or iCa^2+^_7.40_ to evaluate the association between clinical variables (breed, age, milking status, disease category, sex) using univariable linear regression analysis. Then, a specific modeling approach was used for determining the relationship between iCa^2+^ and selected plasma or serum variables. The distribution of the different continuous predictors was visually assessed and tested using the Shapiro‐Wilk test. Small deviations were observed from normality but were not significantly improved after either log transformation or Box‐Cox transformation. For this reason, continuous variables were described as median, range and interquartile range as previously reported.^4^ After evaluating the risk of multicollinearity using Spearman rho analysis, when a pair of potential predictors had a correlation ≥0.7 only 1 of them was kept as a potential regressor. Univariable analyses then were performed to detect variables associated with *c*iCa^2+^ or *c*iCa^2+^_7.40_. Predictors with univariable *P* values <.10 were kept for the multivariable regression analysis.

Two different models were built using *c*iCa or *c*iCa_7.40_ as the dependent variable and other biochemical predictors using the general framework:

$$\mathrm{iCa}\;\text{or}\;{\mathrm{iCa}}_{7.40}=\alpha_{0}+\sum_{i=1}^{n}\alpha_{i}\times \left(X_{i}\right)+\varepsilon ,$$

where *α* are the coefficients associated with the covariate vector *X* of the different biochemical variables and *ε* the model residuals. A manual backward stepwise strategy was used after including all significant variables found in univariable analysis and removing variables with *P* values ≥.05. The final model was the first model with all remaining significant variables (ie, *P* < .05). Specific attention was paid to the assumptions of the linear regression model (ie, homoscedasticity and normally‐distributed residuals). The residual distribution was observed visually and considered adequate if it appeared to be normally distributed. Outliers were defined using QQplot, and standardized residuals were specifically investigated and their impact assessed after removing them from the dataset. This impact was judged negligible and therefore no specific model adjustment was performed. The fit of the models was assessed using adjusted R‐squared. The relative importance of each individual independent variable in the model was assessed determining the partial R‐squared of the variable as well as global R‐squared of the model and variation inflation factor (VIF). Results were considered significant when *P* values were <.05.

## 3 RESULTS

### 3.1 Study population

Four cattle breeds were included: Simmental or Simmental cross‐breed (n = 238), Swiss Brown or Swiss Brown cross‐breed (n = 14), Holstein or Holstein cross‐breed (n = 11) and Jersey cattle (n = 2). There were 240 female and 25 male cattle, ranging from 1 to 12 years in age (median, 3.6 years). Ninety‐three animals were <3 years old (35%), 90 were between 3 and <5 years old (34%) and 82 cows were >5 years old (31%). A total of 184 of 240 (76.7%) cows were in lactation and 26 cows were ≤3 weeks postpartum.

### 3.2 Selected blood acid‐base and serum biochemical results

The range, median, and interquartile range of venous blood pH, *c*iCa^2+^, plasma *c*Cl, *c*Na, *c*K, and *c*tCa, and total protein, albumin, and globulin concentrations are presented in Table 1. The intra‐assay CVs of the Radiometer ABL80 analyzer for venous blood pH and *c*iCa^2+^, *c*Cl, *c*Na, and *c*K were 0.2%, 0.4%, 0.7%, 0.4%, and 0.3%, respectively. The inter‐assay CVs for the *c*tCa, and total protein, and albumin concentrations were 3.4%, 4.3%, and 5.2%, respectively.

**TABLE 1:** The range, median, and interquartile range of venous blood pH, ionized calcium, plasma chloride, sodium, potassium, serum total calcium, total protein, albumin, and globulin concentrations in 265 sick adult cattle.

Variable	Range (median)	Interquartile range
Blood iCa^2+^ (mmol/L)	0.41‐1.40 (1.10)	1.00‐1.17
Serum tCa (mmol/L)	1.11‐2.67 (2.03)	1.87‐2.16
Blood pH	7.16‐7.69 (7.48)	7.44‐7.52
Plasma chloride (mmol/L)	45‐113 (97)	90‐102
Plasma sodium (mmol/L)	118‐152 (139)	135‐142
Plasma potassium (mmol/L)	1.83‐5.36 (3.64)	3.21‐3.91
Plasma SID_3_ (mEq/L)	25.7‐76.8 (45.9)	42.5‐50.4
Serum total protein (g/dL)	3.9‐10.6 (7.0)	6.3‐7.8
Serum albumin (g/dL)	1.7‐4.4 (3.2)	2.8‐3.4
Serum globulin (g/dL)	1.3‐8.1 (3.8)	3.1‐4.6

Abbreviations: iCa^2+^, ionized calcium; SID_3_, strong ion difference; tCa, total calcium.

### 3.3 Clinical diagnoses in the study population

Cattle were diagnosed with various diseases (gastrointestinal disorders [n = 164; abomasitis, abomasal impaction, abomasal ulcer, acute ruminal lactic acidosis, cecal dilatation, chronic rumen acidosis, enteritis, ileus, intestinal invagination, omasum constipation, right‐displaced abomasum, simple indigestion, salmonellosis, traumatic reticuloperitonitis or perireticular abscess, vagus indigestion, peritonitis], respiratory disorders [n = 48; aspiration pneumonia, laryngitis, laryngeal edema, pleuropneumonia, pneumonia, tracheal mass], systemic disorders [n = 21; theileriosis, anaplasmosis, malignant catarrhal fever, bovine ephemeral fever, sepsis, mucosal disease, hypovitaminosis A, cerebrocortical necrosis], acute mastitis [n = 7], reproductive disorders [n = 6; acute metritis, retained fetal membranes], metabolic disorders [n = 12; hepatic lipidosis, hypomagnesemia, ketosis, milk fever], and others [n = 7; traumatic pericarditis, laminitis, cystitis]).

**FIGURE 1:**
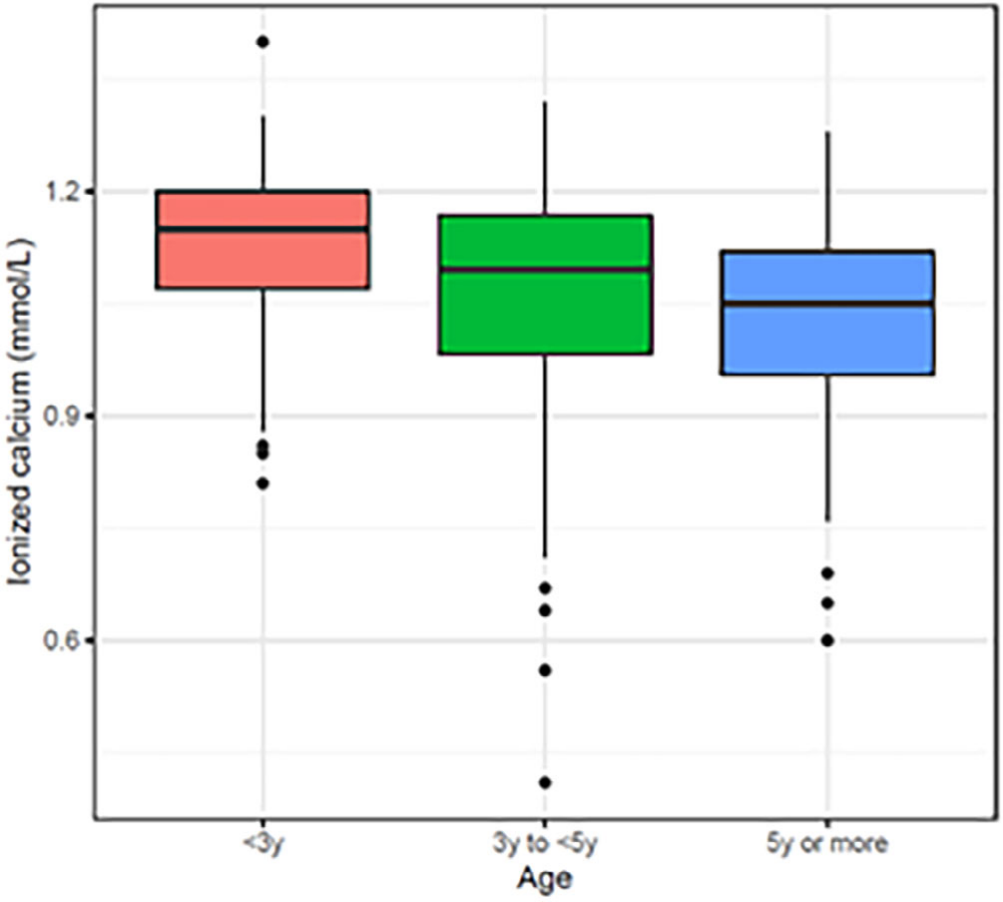
The distribution of blood ionized calcium (iCa^2+^) concentration by age category in sick adult cattle.

The univariable analyses between iCa^2+^, iCa_7.40_ and clinical predictors are presented in Table 2. Blood iCa^2+^ concentration and *c*iCa_7.40_ were negatively associated with age, with older cows having lower *c*iCa^2+^ results than cows <3 years of age (Figure 1). Differences also were observed based on the type of disease and sex with males having higher *c*iCa^2+^ than females (Table 2).

**TABLE 2:** The results of univariable analyses between concentrations of ionized calcium (iCa^2+^), iCa^2+^_7.40_ and clinical predictors in 265 sick adult cattle.

Unadjusted blood iCa^2+^ concentration				Adjusted blood iCa^2+^ concentration to pH = 7.40				
Characteristic	N	Beta	95% CI	*P*‐value	Characteristic	Beta	95% CI	*P*‐value
Breed	265			.64	Breed			.65
Simmental	238 (89.8%)	–	–		Simmental	–	–	
Brown Swiss	14 (5.3%)	0.01	−0.07 to 0.09	1	Brown Swiss	0.02	−0.06 to 0.09	.97
Holstein	11 (4.1%)	0.03	−0.06 to 0.12	.89	Holstein	0.02	−0.07 to 0.11	.97
Jersey	2 (0.8%)	−0.11	−0.31 to 0.09	.73	Jersey	−0.11	−0.31 to 0.09	.69
Age	265			<.001	Age			<.001
<3y	93 (35.0%)	–	–		<3y	–	–	
3y to <5y	90 (34.0%)	−0.06	−0.10 to −0.02	.003	3y to <5y	−0.06	−0.10 to −0.02	.002
5y or more	82 (31.0%)	−0.08	−0.13 to −0.04	<.001	5y or more	−0.07	−0.12 to −0.03	<.001
Diseases	265			<.001	Diseases			<.001
Gastrointestinal	164 (61.9%)	–	–		Gastrointestinal	–	–	
Respiratory	48 (18.1%)	0.08	0.04 to 0.13	.01	Respiratory	0.08	0.03 to 0.12	.01
Systemic	21 (8%)	0.08	0.01 to 0.14	.24	Systemic	0.06	−0.01 to 0.12	.56
Metabolic	12 (4.5%)	0.06	−0.02 to 0.14	.79	Metabolic	0.04	−0.04 to 0.13	.93
Mastitis	7 (2.6%)	0.01	0.04 to 0.25	.11	Mastitis	0.15	0.05 to 0.26	.07
Others	7 (2.6%)	0.01	−0.10 to 0.11	1	Others	0.01	−0.10 to 0.11	1
Reproductive	6 (2.3%)	0.04	−0.07 to 0.16	.99	Reproductive	0.05	−0.06 to 0.16	.98
Milking status	240			.74	Milking status			.68
No	56 (23.3%)	–	–		No	–	–	
Yes	184 (76.7%)	−0.01	−0.05 to 0.04		Yes	−0.01	−0.05 to 0.03	
Gender	265			.01	Gender			.05
Female	240 (90.6%)	–	–		Female	–	–	
Male	25 (9.4%)	0.08	0.02 to 0.13		Male	0.08	0.00 to 0.12	

Abbreviations: CI, confidence interval; iCa^2+^, ionized calcium.

### 3.4 Evaluation of serum total calcium concentration diagnostic performance

In our population of 265 cattle, 96 (36.2%), 159 (60.0%), and 10 (3.8%) were classified as hypocalcemic, normocalcemic, and hypercalcemic on the basis of *c*iCa^2+^, respectively, whereas 113 (42.6%), 151 (57.0%), and 1 (0.4%) cattle were hypocalcemic, normocalcemic, and hypercalcemic on the basis of *c*tCa, respectively. Sensitivity, Sp, NLR, and PLR for *c*tCa measurements for the diagnosis of abnormal *c*iCa^2+^ are presented in Table 3. To detect blood ionized hypocalcemia, Se of *c*tCa measurements with reference range of 2.00‐2.60 mmol/L was 72.9% (95% confidential interval, 62.9‐81.5). The diagnostic discordance between *c*tCa and *c*iCa^2+^ measurements was 30.2%. Diagnostic discordance of *c*tCa measurements in cattle with blood ionized hypocalcemia was 27.1%.

**TABLE 3:** Sensitivity, specificity, and positive and negative likelihood ratios of serum total calcium measurements for diagnosis of abnormal blood ionized calcium concentrations.

Reference interval for tCa	Sensitivity (95% CI)	Specificity (95% CI)	PLR (95% CI)	NLR (95% CI)
2.00–2.60 mmol/L (n = 265)	66.0% (56.2%–75.0%)	72.3% (64.7%–79.1%)	2.39 (1.79‐3.18)	0.47 (0.35‐0.62)

*Note*: Reference interval for serum total calcium concentrations were shown in the table.

Abbreviations: CI, confidence interval; NLR, negative likelihood ratio; PLR, positive likelihood ratio; tCa, serum total calcium.

### 3.5 Relationships among venous blood pH, chloride, sodium, potassium, serum total calcium, albumin, globulin, and blood ionized calcium

**FIGURE 2:**
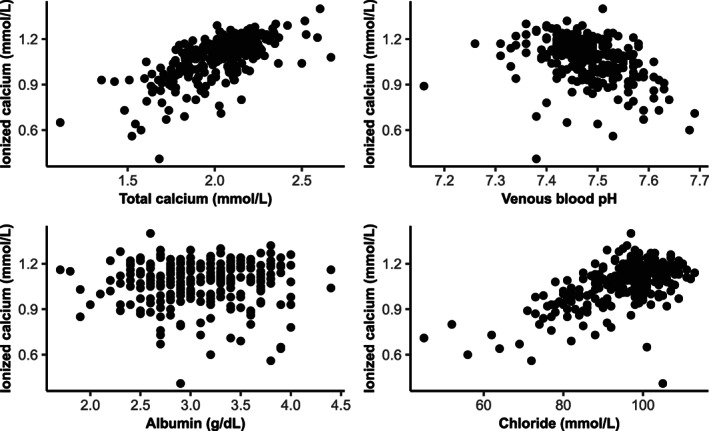
Scatterplots of serum total calcium concentrations, serum albumin concentrations, venous blood pH, and plasma chloride concentrations with blood ionized calcium concentrations. Blood ionized calcium concentration is presented on *y*‐axis.

**FIGURE 3:**
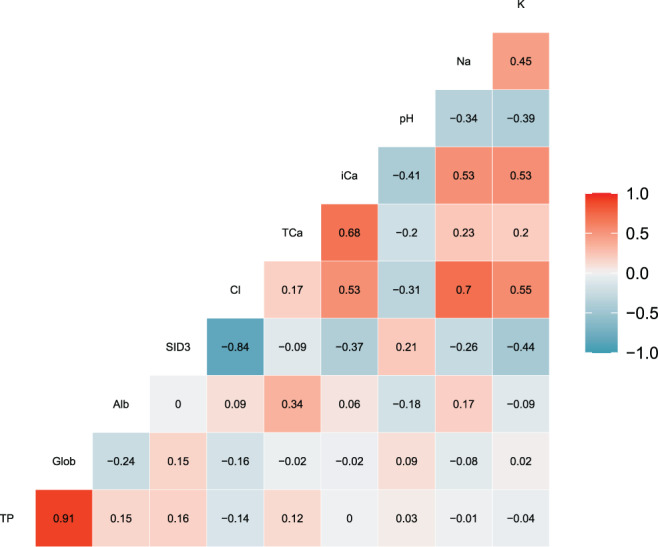
Spearman correlations among *c*iCa^2+^, venous blood pH, serum total calcium concentrations, serum albumin concentrations, serum globulin concentrations, serum total protein concentrations, plasma chloride concentrations, plasma potassium concentrations, plasma sodium concentrations, and plasma strong ion difference (SID_3_).

The individual relationships between *c*iCa^2+^ and *c*tCa, and serum albumin concentrations, venous blood pH, and plasma *c*Cl are shown in Figure 2. Correlations among *c*iCa^2+^, venous blood pH, *c*tCa, serum albumin concentrations, serum globulin concentrations, serum total protein concentrations, plasma cCl, plasma cK, and plasma cNa are presented in Figure 3. The correlations between *c*iCa^2+^ and *c*tCa, plasma cCl, and cK were positive whereas, the correlations between *c*iCa^2+^ and blood pH and plasma SID_3_ were negative. The univariable analysis and multivariable analysis results of blood variable associations with *c*iCa^2+^ and *c*iCa^2+^_7.40_ are presented in Tables 4 and 5, respectively. The median percentage of blood iCa^2+^ fraction in total serum calcium was 53.8%, ranging from 24.3% to 68.8%. The correlation between predictors showed that sodium and chloride, as well as globulin and total protein were highly correlated (r = 0.70 and 0.91, respectively). Therefore, only chloride and globulin were kept for regression analysis. The VIF analysis did not identify problems of multicollinearity (VIF < 5).

**TABLE 4:** Results of univariable analyses of the association of selected blood, plasma, or serum analytes with blood ionized calcium (iCa^2+^) concentration in 265 adult cattle with different diseases.

Unadjusted blood iCa^2+^				Blood iCa^2+^ concentration adjusted to pH = 7.40			
Variable	Beta	95% CI	*P*‐value	Variable	Beta	95% CI	*P*‐value
Potassium	0.14	0.11 to 0.17	<.001	Potassium	0.12	0.09 to 0.15	<.001
Chloride	0.01	0.01 to 0.01	<.001	Chloride	0.01	0.01 to 0.01	<.001
Blood pH	−0.76	−1.00 to −0.52	<.001	Blood pH	−0.25	−0.50 to 0.00	.05
tCa	0.44	0.38 to 0.50	<.001	tCa	0.44	0.38 to 0.49	<.001
Globulin	0	−0.02 to 0.01	.55	Globulin	0	−0.02 to 0.01	.78
Albumin	0.01	−0.03 to 0.04	.77	Albumin	−0.01	−0.04 to 0.03	.75
Hematocrit	−0.01	−0.01 to 0.00	<.001	Hematocrit	−0.01	−0.01 to 0.00	<.001

Abbreviations: CI, confidence interval; iCa^2+^, ionized calcium; tCa, serum total calcium.

**TABLE 5:** The results of multivariable analysis of the association between selected blood, plasma, or serum analytes of blood ionized calcium (iCa^2+^) concentration with blood ionized calcium concentration in 265 adult cattle with different clinical disorders.

Unadjusted blood iCa^2+^						Adjusted blood iCa^2+^ concentration to pH = 7.40					
Variable	Coefficient	SE	*P* value	Partial *R* ^2^	VIF	Variable	Coefficient	SE	*P* value	Partial *R* ^2^	VIF
Intercept	1.32	0.586	.03	–	–	Intercept	−2.784	0.600	<.001	–	–
tCa (mmol/L)	0.409	0.022	<.001	0.393	1.17	tCa (mmol/L)	0.426	0.022	<.001	0.422	1.17
Chloride (mmol/L)	0.005	0.001	<.001	0.208	1.79	Chloride (mmol/L)	0.005	0.001	<.001	0.207	1.79
Potassium (mmol/L)	0.033	0.011	.003	0.104	1.99	Potassium (mmol/L)	0.036	0.012	.003	0.085	1.99
Albumin (g/dL)	−0.060	0.010	<.001	0.008	1.19	Albumin (g/dL)	−0.064	0.010	<.001	0.014	1.19
Blood pH	−0.200	0.075	.01	0.040	1.30						
			*R* ^2^	0.752					*R* ^2^	0.730	
			Adjusted *R* ^2^	0.748					Adjusted *R* ^2^	0.725	

Abbreviations: iCa^2+^, ionized calcium, tCa, serum total calcium; VIF, variation inflation factor.

## 4 DISCUSSION

The ability of *c*tCa measurements to accurately predict abnormal *c*iCa^2+^ was assessed in adult cattle with a variety of clinical diseases. Our findings indicated that *c*tCa measurements failed to accurately predict *c*iCa^2+^ in ill adult cattle. Serum tCa concentration measurements accounted for only 42% of *c*iCa^2+^_7.40_ variance. Plasma *c*Cl, and *c*K were also predictive of *c*iCa^2+^_7.40_, accounting for an additional 21% and 9% of the variance, respectively.

Serum total calcium concentration measurements usually have been used to assess calcium status in bovine practice although Se, Sp, PLR, NLR, and diagnostic discordance of *c*tCa measurements against *c*iCa^2+^ measurements have not been previously calculated in cattle with different clinical disorders. In our study, the diagnostic discordance between *c*tCa and *c*iCa^2+^ was 30.2%, indicating that *c*tCa measurements did not correctly predict iCa^2+^ status in at least 30.2% of cattle with different disorders. Similarly, diagnostic discordance of *c*tCa in dogs previously was determined as 27.0%^16^ or 18.5%. In the latter study, 80% of dogs had normal *c*iCa^2+^ concentrations.^17^ In our study, the diagnostic discordance between *c*tCa measurements and *c*iCa^2+^ measurements was high in sick adult cattle with blood ionized hypocalcemia (27.1%). The reference range of 2.00‐2.60 mmol/L for *c*tCa showed poor sensitivity (66.0%). The low PLR values (2.39) showed that most cattle with abnormal blood iCa^2+^ status were incorrectly identified as normocalcemic by *c*tCa measurements. In our study, NLR > 0.1 (0.47) for *c*tCa measurements showed that *c*tCa measurement is not a good test to rule out a diagnosis of abnormal *c*iCa^2+^. Overall, our results suggest that *c*tCa measurements for correctly predicting *c*iCa^2+^ had poor diagnostic performance in sick cattle. From a clinical view, the use of *c*tCa measurements instead of *c*iCa^2+^ determination may cause misclassification of calcium status in cattle with clinical disorders. Thus, *c*iCa^2+^ measurements should be performed whenever available.

Data regarding the reference interval of *c*iCa^2+^ in cattle is limited. The reference interval of *c*iCa^2+^ used in our study was taken from another study.^11^ In that study, 50 healthy Swedish red and white breed cows were used to determine a reference range for serum iCa^2+^ concentration using a different calcium ion analyzer from that used in our study. Additional studies are necessary to determine the reference range of *c*iCa^2+^ in healthy cattle populations using calcium ion analyzers available today. Different reference ranges of *c*tCa in cattle have been used in bovine clinics. The reference interval of *c*tCa used in our study was taken from a textbook.^13^ The same reference range for *c*tCa has been used in the clinic where the study was performed. Moreover, subclinical hypocalcemia in periparturient dairy cows was defined as *c*tCa <2.00 mmol/L in some studies.^18^, ^19^ Similarly, the lower value of the reference interval for *c*tCa was 2.00 mmol/L in our study.

Blood iCa^2+^ concentration measurements were performed within 10 minutes of blood collection, but serum samples were stored at −20°C for *c*tCa measurements until analyzed within 7 days of collection. It was shown that whole blood samples may be stored at least 14 days at 4°C in plain or lithium heparin tubes with no changes in *c*tCa concentrations in cattle.^20^ Storage of serum at −80°C had no effect on *c*tCa for up to 12 months in cattle.^20^ The results of that study indicate that storage condition and timing of serum samples did not have an effect on *c*tCa measurements in our study.

Blood iCa^2+^ concentration in critically ill neonatal calves was mainly dependent on plasma tCa concentration, plasma *c*Cl, and venous blood pH.^4^ Total calcium, serum *c*Cl, and albumin concentrations were the most important variables affecting *c*iCa^2+^ in dogs and cats with different clinical disorders where the effect of blood pH on *c*iCa^2+^ was not evaluated.^17^, ^21^ The multivariable regression model in our study provided valuable information on the relationships between selected variables and *c*iCa^2+^. Our results indicated that *c*tCa, plasma *c*Cl, and *c*K were positively associated with *c*iCa^2+^ in adult cattle affected by different diseases whereas a negative association was found between serum albumin concentration and *c*iCa^2+^.

Apart from *c*tCa, plasma *c*Cl had the strongest association with *c*iCa^2+^ in adult cattle in our study with different clinical disorders. An overlooked physicochemical phenomenon is that an increase in serum *c*Cl directly increases the number of chloride ions bound to bovine albumin,^22^ and the increased chloride binding displaces calcium from adjacent electrostatic binding sites, thereby increasing serum ionized Ca^2+^ concentration.^23^ The positive association between blood *c*Cl and *c*iCa^2+^ in our study is consistent with previous findings obtained from dogs, cats, and neonatal calves, where *c*iCa^2+^ increased as serum or plasma *c*Cl increased.^4^, ^17^, ^21^ Chloride ions are bound in a salt‐type manner to positively charged guanidium and ε‐amino groups in albumin despite the net negative charge of albumin at physiologic pH,^24^ with 3 chloride ions being electrostatically bound to bovine albumin at physiologic pH.^22^ The effect of chloride binding to albumin on *c*iCa^2+^ does not appear to be because of the effect of a decrease in plasma pH because of a decrease in SID because plasma *c*Cl was positively correlated with plasma *c*Na (r = 0.70) and plasma *c*K (r = 0.55) in our study, indicating minimal change in plasma SID and therefore plasma pH because of large changes in plasma *c*Cl. Similar to our study, the effect of chloride ions on *c*iCa^2+^ was observed to be independent of its effect on pH in critically ill neonatal calves.^4^ In accordance with our results, diet‐induced increases in plasma *c*Cl by feeding an acidogenic ration in dairy cows during early lactation was accompanied by an increase in *c*iCa^2+^ but no change *c*tCa.^25^ Moreover, in cows with parturient paresis, IV administration of CaCl_2_ resulted in higher serum iCa^2+^ concentrations than when the same amount calcium was administered as calcium borogluconate despite the fact that no difference was observed in *c*tCa.^12^ Taken together, these findings indicate that clinical evaluation of *c*tCa in sick adult cattle would benefit from simultaneous evaluation of blood chloride concentration, because of the direct effect of plasma *c*Cl on iCa^2+^ binding to albumin.

Current recommendations are that for clinical application, *c*iCa^2+^ and blood pH should be measured simultaneously and within 15 minutes in samples kept at room temperature or 4 hours in samples collected in iced water, and *c*iCa^2+^ reported as actual and adjusted to a pH of 7.40.^26^ Correction for pH compensates for preanalytical pH alterations associated with incorrect anaerobic handling of the sample and loss of CO_2_ through the wall of the polypropylene syringe during storage. A decrease in pH most likely increases *c*iCa^2+^ through pH‐induced changes in net imidazole charge in specific histidine groups in albumin, resulting in a localized change in charge distribution that decreases iCa^2+^ binding. In multivariable regression analysis, comparison of actual pH vs pH‐corrected values for iCa^2+^ indicated a pH‐independent effect of chloride binding to albumin on *c*iCa^2+^ in that changes in chloride concentration had the same proportional effect on *c*iCa^2+^ whether *c*iCa^2+^ was actual or corrected to a pH of 7.40.

Taken together, our results and that of a previous study in calves^4^ indicate that clinically relevant changes in plasma *c*Cl exert a greater effect on *c*iCa^2+^ than clinically relevant changes in plasma pH.

A negative association between venous blood pH and *c*iCa^2+^ was identified in critically ill neonatal calves.^4^ Moreover, venous blood pH had the second largest effect after plasma tCa concentrations on *c*iCa^2+^, and venous blood pH explained 19% of the variation of *c*iCa^2+^ in those calves.^4^ Univariable regression in our study also indicated a negative association between venous blood pH and *c*iCa^2+^ in adult cattle, but the association was weak in the multivariable regression model. Our results suggest that the effect of venous blood pH on *c*iCa^2+^ in sick adult cattle is lower than that previously appreciated.^4^ The median venous blood pH in ill neonatal calves was 7.31, ranging from 6.9 to 7.54 in that study^4^ whereas the median of venous blood pH in sick adult cattle was 7.48, ranging from 7.16 to 7.65 in our study. Moreover, in our study, venous blood pH results of 180 of 265 cattle were >7.46. Higher venous blood pH of sick adult cattle might be an explanation for this finding.

Plasma *c*K was positively associated with *c*iCa^2+^ in sick adult cattle in our study, accounting for 9% of the variation in *c*iCa^2+^. The range for blood *c*K was 1.8‐5.4 mmol/L (median, 3.6 mmol/L) in adult cattle. Plasma *c*K was not significantly associated with *c*iCa^2+^ in critically ill neonatal calves.^4^ In that study, the range for blood *c*K was 2.1‐11.5 mmol/L (median, 4.6 mmol/L). Serum *c*K was positively associated with *c*iCa^2+^ in dogs and cats, but the association was weak relative to that of tCa, Cl, and albumin^17^, ^21^ Differences in blood *c*K may account for the different associations between plasma *c*K and *c*iCa^2+^ between ill neonatal and sick adult cattle. More likely, the positive association between potassium and calcium concentrations in sick adult cattle reflects the effect of inappetence and decreased calcium and potassium intake, with calcium and potassium losses being increased in lactating cattle. Concurrent decreases in serum chloride, potassium, and calcium concentrations are common clinicopathologic findings in dehydrated or endotoxemic adult cattle, particularly during the postpartum period. Our results imply that administration of fluids containing chloride and potassium during fluid therapy to adult sick cattle may result in increases in *c*iCa^2+^ in clinical practice.

In our study, serum albumin concentration was significantly associated with *c*iCa^2+^, but serum albumin concentration accounted for only 1% of the variation in *c*iCa^2+^ of adult cattle with different clinical disorders. This finding indicates that clinical evaluation of *c*tCa in sick adult cattle does not require simultaneous evaluation of serum albumin concentration. In critically ill calves, univariate regression analyses showed that serum albumin concentration had a significant but weak effect relative to *c*tCa, venous blood pH, and *c*Cl on *c*iCa^2+^. In that study, serum albumin concentration was not a significant predictor of *c*iCa^2+^ in a stepwise multivariable regression model.^4^ Serum albumin concentration had an influence on *c*iCa^2+^ in dogs^17^ and cats.^21^ Species differences in the number of calcium binding sites on albumin and net albumin charge could play a role in the observed differences.

The iCa^2+^ percentage of *c*tCa has been investigated primarily in clinically healthy cattle and constituted 51% in 141 clinically healthy cows.^7^ In that study, calcium concentrations were measured within 2 hours after blood collection.^7^ The percentage of serum iCa^2+^ was determined as 43% in clinically healthy cows in different stages of lactation where most samples were analyzed within 24 hours, but some measurements were performed within 4 days.^9^ Mean plasma iCa^2+^ percentage was 57% at parturition and then decreased to 53% at peak lactation in clinically healthy Holstein and Jersey cows.^27^ Use of serum or plasma samples for the determination iCa^2+^ and time from sample collection to analysis might have influenced the blood iCa^2+^ percentage in the studies noted above. Blood iCa^2+^ % in *c*tCa changed from 49.6% to 47.2% depending on dietary cation‐anion difference at prepartum period in clinically healthy cows. In that study, blood iCa^2+^ measurements were made within 30 minutes of sampling.^25^ In another study, blood iCa^2+^ % in *c*tCa was approximately 52% at prepartum day 3 and then increased to approximately 54% at parturition in cows fed with a dietary cation‐anion difference of −7 where *c*iCa^2+^ was measured within 1 hour of sample collection.^28^ The range of blood iCa^2+^ percentage was 35 to 61% in 950 critically ill neonatal calves.^4^ Blood iCa^2+^ concentration and *c*tCa measurement methods in the previous 3 studies were similar to those of our study. In our study, the range of blood iCa^2+^ percentage in sick adult cattle (24%‐69%) was wider than that of critically ill neonatal calves.^4^ Moreover, the median *c*iCa^2+^ of sick adult cattle was higher than that of sick neonatal calves (54% vs 47%). It has been suggested that approximately 50% of calcium in bovine blood exists in the ionized form. This assumption has been based on previous studies performed on clinically healthy cattle. In our study, blood iCa^2+^ percentages in *c*tCa of 100 of 265 cattle were >55%. The results of our study indicated that the assumption noted above might not be valid for sick cattle. Moreover, we observed lower median iCa^2+^ % in *c*tCa in cattle with gastrointestinal disorders than in cattle with other system diseases but its median value still was >50% in these cattle.

A potential limitation of our study was the failure to measure plasma biochemical variables that have been associated in other studies with *c*iCa^2+^. Serum nonesterified fatty acids and beta‐hydroxybutyric acid concentrations are negatively correlated with iCa^2+^ % in clinically healthy periparturient dairy cows.^29^ Serum magnesium and plasma L‐lactate concentrations are associated with *c*iCa^2+^ in critically ill neonatal calves.^4^ Serum creatinine, urea nitrogen, cholesterol, and triglyceride concentrations are associated with *c*iCa^2+^ in dogs and cats.^17^, ^21^ It is well known that calcium metabolism disorders are an important problem during the postpartum period. In our study, 26 out of 240 cows were within 3 weeks postpartum. This limited number of early lactating cows is partly associated with the selection criteria which excluded cows that previously received calcium treatment to limit interference of treatment with study findings. Therefore, additional studies appear indicated, especially in periparturient dairy cows, to investigate the association of analytes such as nonesterified fatty acids, beta‐hydroxybutyric acid, phosphorus, and magnesium on *c*iCa^2+^.

In conclusion, *c*tCa measurements failed to accurately predict *c*iCa^2+^ status in ill adult cattle. The *c*iCa^2+^ had a positive relationship with *c*tCa, plasma *c*Cl and *c*K, and with *c*tCa and plasma *c*Cl, accounting for 63% of the variation on *c*iCa^2+^. Our results showed that venous blood pH and serum albumin concentration are not significant predictors of *c*iCa^2+^ in sick adult cattle. Accurate clinical evaluation of calcium status using *c*tCa measurements in adult cattle with clinical disorders would benefit from simultaneous evaluation of *c*Cl and possibly *c*K because hypochloremia, hypokalemia, and hypocalcemia are common concurrent electrolyte disorders in sick adult cattle, particularly cattle with primary gastrointestinal tract diseases.

## CONFLICT OF INTEREST DECLARATION

Sébastien Buczinski serves as Consulting Editor for Experimental Design and Statistics for the Journal of Veterinary Internal Medicine. He was not involved in review of this manuscript. No other authors declare a conflict of interest.

## OFF‐LABEL ANTIMICROBIAL DECLARATION

Authors declare no off‐label use of antimicrobials.

## INSTITUTIONAL ANIMAL CARE AND USE COMMITTEE (IACUC) OR OTHER APPROVAL DECLARATION

Approved by the Firat University Ethics Committee on Animal Experimentation, Protocol Number: 2019/123, Decision Number: 179.

## HUMAN ETHICS APPROVAL DECLARATION

Authors declare human ethics approval was not needed for this study.
